# Pendulum swings from hypo- to hyperthyroidism: thyrotoxicosis after severe hypothyroidism following neck irradiation in a patient with a history of Hodgkin’s lymphoma

**DOI:** 10.1186/s13044-016-0030-1

**Published:** 2016-01-15

**Authors:** Krzysztof Lewandowski, Katarzyna Dąbrowska, Jacek Makarewicz, Andrzej Lewiński

**Affiliations:** Department of Endocrinology and Metabolic Diseases, Polish Mother’s Memorial Hospital - Research Institute, Lodz, Poland; Department of Endocrinology and Metabolic Diseases, Medical University of Lodz, Lodz, Poland; Department of Nuclear Medicine and Oncological Endocrinology, Zgierz District Hospital, Zgierz, Poland

**Keywords:** Thyrotoxicosis, Graves’ disease, Neck irradiation, Thyroid antibodies

## Abstract

**Background:**

A change in a thyrometabolic state from severe hypothyroidism to thyrotoxicosis is very uncommon, but possible in some circumstances.

**Case presentation:**

A 27-year old female presented with clinical and biochemical thyrotoxicosis with a previous history chemo- and radiotherapy (including the neck region) for a Hodgkin’s lymphoma (at the age of 18). At the age of 20 this was followed by severe hypothyrodism [TSH > 100 μIU/mL (reference range: 0.27–4.2)]. She was stated on L-thyroxine, but the dose was later reduced and subsequently discontinued. She had significantly elevated titres of both anti-thyroid peroxidase antibodies and anti-TSH-receptor antibodies throughout the course of disease. Thyroid scintigraphy revealed a normal and homogenous iodine uptake.

**Conclusions:**

We suspect that a gradual switch from thyroid-blocking to thyroid-stimulating antibodies resulted in development of an overt thyrotoxicosis, possibly with a contributory effect of neck irradiation on her autoimmune status.

## Background

Though severe hypothyroidism usually requires life-long treatment with L-thyroxine, in rare circumstances a remission of such state, or even a switch into thyrotoxicosis could be observed. Below we present a case of severe hypothyroidism followed by thyrotoxicosis in a patient with a previous history of chemo- and radiotherapy (including a neck irradiation) for a Hodgkin’s lymphoma.

## Case presentation

A 27-year old female presented with clinical and biochemical thyrotoxicosis (TSH - 0.01 μIU/mL (ref. range: 0.27–4.2 μIU/mL); free thyroxine (FT_4_) - 1.58 ng/dL (ref. range 0.98–1.63 ng/dL); free triiodothyronine (FT_3_) - 4.56 pg/mL (ref. range 2.6–4.4 pg/mL). Clinical examination revealed tachycardia about 100 beats/min and no obvious goitre. Autoimmune profile was suggestive of Graves’ disease [anti-TSH-receptor antibodies (aTSHR) - 16.69 IU/L (ref. 0–1.75), anti-thyroid peroxidase antibodies (aTPO) - 1780 IU/mL (ref.: 0–34 IU/mL)]. The patient had a history of Hodgkin’s lymphoma, diagnosed and treated with chemo- and radiotherapy (including the neck) at the age of 18. At the age of 20 she developed severe hypothyroidism (TSH > 100 μIU/ml), though with high titres of both aTPO (150 IU/mL) and aTSHR (37.56 IU/mL) antibodies. Thyroid function tests normalised after treatment with L-thyroxine (100 μg o.d.). At the age of 26 she became “anxious” and experienced “heart palpitations”. She was found to have suppressed TSH, that remained suppressed even when the dose of L-thyroxine was reduced and then discontinued. After further four months she was found to have raised FT_3_ (see above). Thyroid scintigraphy revealed a normal and homogenous iodine uptake (41 %). The patient responded very well to treatment with low dose thiamazole (10 mg od, later 5 mg o.d.) that was discontinued after about 12 months. About a year later (while 16-week pregnant) she was off medication and had mild subclinical thyrotoxicosis (TSH - 0.035 μIU/mL, FT_4_ - 1.04 ng/dL, FT_3_ - 3.09 pg/mL) and raised aTSHR antibodies, albeit at lower titre than before (7.87 IU/L).

According to the literature data thyroid dysfunction is one of the most common abnormalities seen after radiotherapy for Hodgkin's disease that includes the neck [1]. Primary hypothyroidism, the most common radiation-induced thyroid dysfunction, appears in 20–30 % patients who had therapeutic radiotherapy administered to the neck region, and this usually occurs within the first 5 years after therapy (peak 2–3 years after treatment) [1]. Irradiation of the thyroid may also increase the risk of Graves' disease (relative risk 7.2–20.4), or Graves' ophthalmopathy, thyroiditis, benign adenomas and thyroid cancer. The aetiology of radiation-induced thyroid dysfunction includes vascular damage, parenchymal cell damage and auto-immune reactions [1]. There are reports, that after neck irradiation Graves’ disease may develop in patients previously receiving thyroxine, and indeed 33 % of the patients with Graves’ hyperthyroidism had received thyroxine before its onset [2]. Therefore thyroid hormone-replacement therapy in patients with hypothyroidism after irradiation of the neck does not eliminate risk of other thyroid abnormalities at a later date. According to some authors, thyroiditis observed in Hodgkin’s disease may be the result of immune regulation disorders in Hodgkin’s disease [3]. Furthermore, a possibility of a change from hypo- to hyperthyroidism typical for Graves’ disease is has been known for some time [4, 5], and was also confirmed in more recent publications [6, 7].

Takeda et al. [8] demonstrated, by measuring changes in cyclic AMP, that thyroid stimulating and thyroid blocking antibodies may coexist within the same patient and their relative activities may change over the course of the disease. Our case illustrates that after neck irradiation, severe hypothyroidism can be followed by thyrotoxicosis. It should be noted that in our patient, both during hypo and hyperthyroidism, aTSHR were elevated. There are two types of aTSHR: thyroid stimulating antibodies (TSAb) and TSH-stimulation blocking antibodies (TBAb). TBAb block TSH-stimulation of the thyroid and cause hypothyroidism. On the other hand, TSAb stimulate the thyroid and result in Graves’ hyperthyroidism. In our opinion, in this case there was a gradual switch from TBAb into TSAb.

In some patients, TSAb and hyperthyroidism develop unexpectedly after hypothyroidism that is caused by TBAb [9]. Fan et al. [10] described a case with documented cycles of hypothyroidism alternating with hyperthyroidism. Also, as mentioned above, some hypothyroid patients after irradiation of the neck, while treated for the Hodgkin's disease, developed hyperthyroidism [2]. A number of mechanisms may be involved in switching from TBAb to TSAb. Significantly, thyroxine treatment in some patients is associated with increase TSAb that - in extreme cases - might lead to development of hyperthyroidism in hypothyroid patients [9], though whether this was a case in our patient is speculative as neck irradiation *per se* is considered to be capable of influencing changes in patients’ immune status.

## Conclusions

Our case demonstrates a possibility of a switch from severe hypothyroidism to thyrotoxicosis, possibly due to a gradual change from TBAb to TSAb in a patient with a previous history of neck irradiation for Hodgkin’s lymphoma. Therefore, thyroid hormone-replacement therapy in patients with hypothyroidism after irradiation of the neck does not eliminate a risk of later thyroid abnormalities.

The case reported above has been preliminarily presented for educational purposes during the conference “Spring School of Thyroidology” organized by the Polish Thyroid Association, 2014 [11].

### Consent

Written informed consent was obtained from the patient for publication of this case report. A copy of the written consent is available for review by the Editor-in-Chief of this journal.
